# Comparative omics profiling reveals differences in biomass, energy production, and vesicle transport between CHO and fast-growing CHL-YN cells

**DOI:** 10.1038/s41598-025-23503-z

**Published:** 2025-11-13

**Authors:** Yu Tsunoda, Rintaro Arishima, Tatiana Boronina, Robert Cole, Noriko Yamano-Adachi, Michael Betenbaugh, Takeshi Omasa

**Affiliations:** 1 Department of Biotechnology, Graduate School of Engineering, The University of Osaka, 2-1 Yamadaoka, Suita, Osaka 565-0871 Japan; 2 Mass Spectrometry and Proteomics Facility, Johns Hopkins University School of Medicine, Baltimore, MD USA; 3 Manufacturing Technology Association of Biologics, 7-1-49 Minatojima-Minami, Kobe, Hyogo 650-0047 Japan; 4 Industrial Biotechnology Initiative Division, Institute for Open and Transdisciplinary Research Initiatives, The University of Osaka, 2-1 Yamadaoka, Suita, Osaka 565-0871 Japan; 5 Department of Chemical and Biomolecular Engineering, Johns Hopkins University, Baltimore, MD USA

**Keywords:** Transcriptomics, Proteomics, Chinese hamster ovary cell, Biological techniques, Biotechnology, Cell biology

## Abstract

Chinese hamster lung (CHL)-YN cells are promising novel hosts for producing therapeutic antibodies with the potential to shorten the research, development, and manufacturing timelines in biopharmaceutical production. CHL-YN cells grow twice as fast as Chinese hamster ovary (CHO) cells, with a doubling time of 8.1 h. These cells possess strong glutamine synthetase activity, allowing them to be cultured in glutamine-free media. In this study, we conducted comparative transcriptomics and proteomics among CHL-YN cells, CHO cells, and lung tissue from Chinese hamster to better understand the global characteristics of CHL-YN cells and determine whether these features are tissue-derived or unique to the cell line. Omics profiling revealed that CHL-YN cells, in contrast to CHO cells, exhibit highly activated processes and pathways related to biomass and energy production, such as translation and biosynthesis of amino acids, but less activated vesicle transport processes, such as Golgi-related vesicle transport. This study highlights distinct characteristics of CHL-YN cells, contributing to streamlined operations, shortened development timelines, and expanded options for selectable cell lines. The findings here could contribute to identifying potential biomarkers and targets for cell engineering toward improving antibody productivity and growth rate in both traditional CHO cells and next-generation host cells, CHL-YN.

**Supplementary Information:**

The online version contains supplementary material available at 10.1038/s41598-025-23503-z.

## Introduction

Chinese hamster (*Cricetulus griseus*) ovary (CHO) cells are the most widely used host cells for biopharmaceutical manufacturing. As of 2022, 89% of therapeutic monoclonal antibodies were produced using them^1^. CHO cells originated from the ovarian tissue of a female Chinese hamster and were first isolated in 1957 by Theodore T. Puck^2^. Numerous researchers have since developed and adapted various CHO cell lines such as CHO-K1, CHO-S, and CHO-DG44^3,4^. Although other mammalian cell lines are also used in biopharmaceutical production, CHO cells are preferred due to their ease of cultivation in serum-free media, ability to perform human-compatible glycosylation, and high amenability to genetic engineering^5,6^. The ease of genetic manipulation has enabled gene amplification systems such as CHO-DG44 cells, greatly contributing to improved productivity of therapeutic antibodies^7,8^. Furthermore, advancements in chemically defined media^9,10^, along with innovations in cultivation processes^11,12^, have led to increased cell concentrations during culture, achieving high yields exceeding 10 g L^−113^. Despite the remarkable success of CHO cells for biopharmaceutical production, their doubling time is approximately 20 h, which is slow compared with non-mammalian hosts^14,15^. This contributes to longer development and production timelines in biopharmaceutical research and manufacturing.

In 2020, Chinese hamster lung (CHL)-YN cells were isolated from the lung of the Chinese hamster and developed as a novel host cell line for biopharmaceutical production^16^. A key feature of CHL-YN cells is their rapid growth; they have demonstrated a doubling time of 8.1 h in a chemically defined medium commonly used for CHO cells, which is far quicker than the 20 h for CHO cells. In terms of monoclonal antibody productivity, CHL-YN cells have been reported to produce a similar amount or more of immunoglobulin G_1_ (IgG_1_) to CHO-K1 cells in a shorter period. For the *N*-glycosylation of IgG_1_, glycan profile analysis using liquid chromatography-tandem mass spectrometry (LC–MS/MS) has shown that IgG_1_ produced by CHL-YN cells exhibits peak patterns entirely similar to those of IgG_1_ produced by CHO-K1 cells^16^. On the other hand, in the case of our previous study, the high-mannose type glycn was detectable in CHL-YN cells^16^. CHL-YN cells exhibit similar characteristics to conventional CHO cells required for host cell applications, while offering more than twice the growth rate, highlighting their potential to shorten research and manufacturing timelines for biopharmaceuticals. However, the extent to which CHL-YN cells differ from CHO cells regarding biological processes, metabolism, and signaling pathways remains unclear. Previous studies reported that glutamine synthetase expression is more than six times higher in CHL-YN cells than in CHO-K1 cells^16^. In previous fed-batch cultures in bioreactors, glutamine concentrations in the medium were initially depleted within the first 2 − 4 days but were observed to subsequently increase, reaching approximately 4–6 mM^17^. Metabolic pathway analyses during fed-batch cultivation revealed distinct temporal changes in intermediate concentrations within the arginine and methionine metabolic pathways between CHO-K1 and CHL-YN cells^17^. These findings suggest that CHL-YN cells differ from CHO cells in their biological processes and metabolic pathways. It is thus necessary to comprehensively characterize the cellular features of CHL-YN cells.

Omics profiling has contributed to a comprehensive understanding of CHO cell biology^18–23^. These approaches have elucidated the underlying biological processes and pathways that are altered in response to variations in process parameters during CHO cell culture, which in turn affect cell growth, productivity, and product quality^24,25^. Omics-based analyses, particularly gene ontology (GO) enrichment and pathway enrichment, have proven powerful in comparative studies across different CHO cell lines, across different species of origin, or between cells with distinct physiological roles. Comparative omics studies between CHO cells and murine or human-derived plasma cells, which are natural antibody producers, have revealed that plasma cells upregulate processes in the endoplasmic reticulum (ER) and membrane trafficking pathways^26,27^. Additionally, comparative proteomics not only among different CHO cell lines but also between CHO cells and various tissue cells derived from Chinese hamster has revealed tissue-specific upregulation of distinct biological processes^28,29^. For instance, the biological processes important for biotherapeutic hosts, including reactive oxidative species metabolism, vesicle transport, and lipid synthesis, were especially enriched in the lung tissues, suggesting that the lung tissues are particularly suitable as an example for comparing omics profiles with cell lines^28^. Such comparisons between tissues and cell lines offer insights into rational cell engineering strategies aimed at conferring specific functions on engineered cell lines. They also suggest the potential of developing new production cell lines from non-ovarian tissues to tailor cellular functions for particular applications.

In this study, we performed comparative transcriptomics and proteomics among CHO cells, CHL-YN cells, and lung tissue derived from Chinese hamsters belonging to the same closed colony as the original Chinese hamster for CHO cells. GO and pathway enrichment analyses were conducted to uncover biological process- and pathway-related differences between CHO and CHL-YN cells. Comparative omics analysis between each cell line and lung tissue revealed shared features associated with their lung tissue origin, as well as characteristics unique to CHL-YN cells. These findings deepen our understanding of CHL-YN cells as a promising novel host for biopharmaceutical production. Moreover, they facilitate the identification of potential biomarkers and targets for cell engineering aimed at improving antibody productivity and growth rate in both traditional CHO cells and next-generation host cells such as CHL-YN.

## Results

### Cell-to-cell and cell-to-tissue similarities

**Fig. 1:**
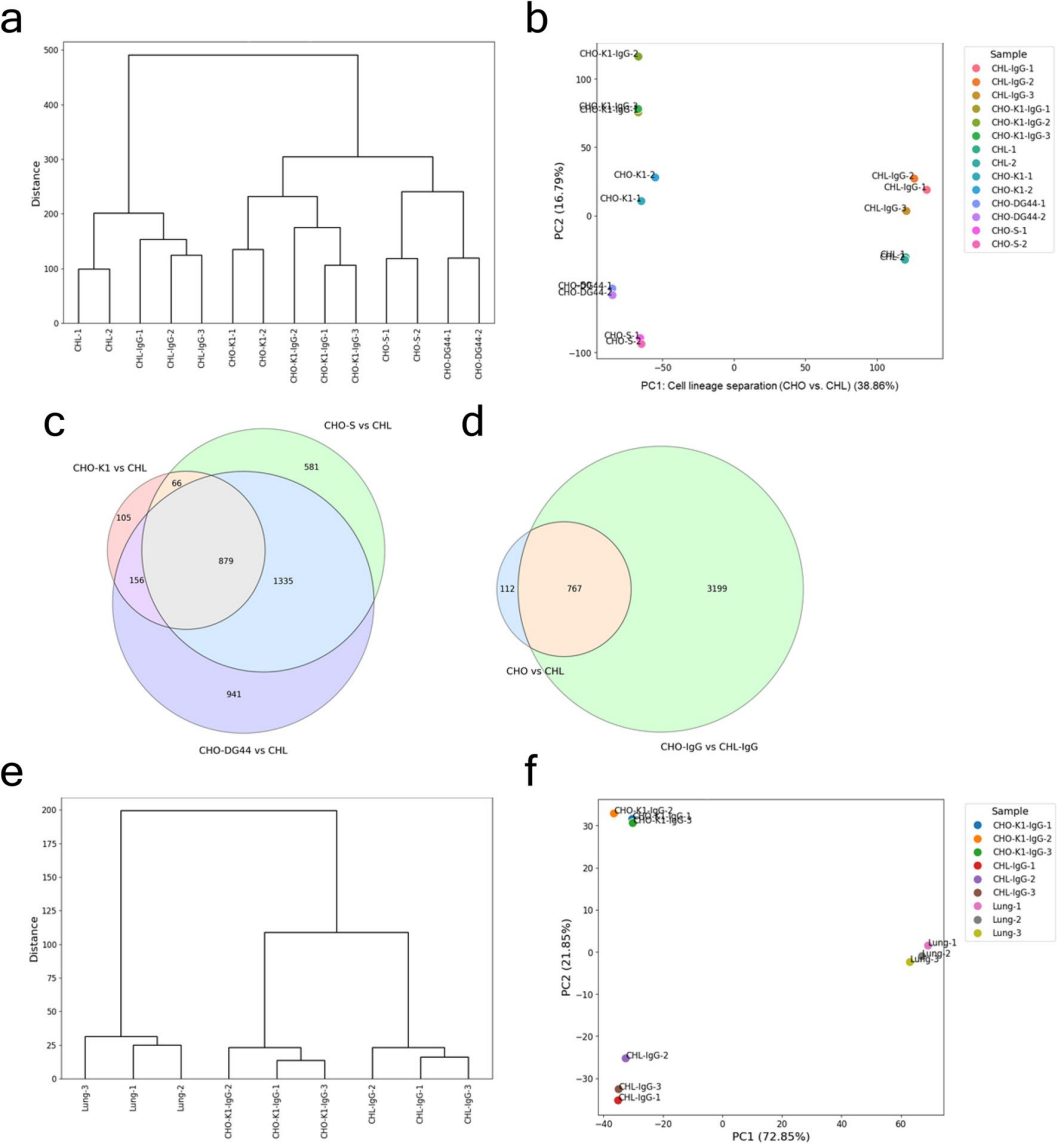
Cell-to-cell and cell-to-tissue similarities. (**a**) Hierarchical clustering and (**b**) principal component analysis (PCA) with scaled values obtained by applying a log_2_ transformation with the addition of 1 to the TPM values of CHO-K1, CHO-DG44, CHO-S, CHO-K1-IgG, CHL, and CHL-IgG cells. (**c**) Venn diagrams of differentially expressed genes (DEGs) between parental CHO and CHL cells. (d) Venn diagrams of DEGs between CHO-K1-IgG and CHL-IgG cells and common DEGs across all pairs of parental CHO and CHL cells. (**e**) Hierarchical clustering and (**f**) PCA with scaled values obtained by applying a log_2_ transformation with the addition of 1 to the protein expression values of CHO-K1-IgG, CHL-IgG cells, and Chinese hamster lung tissues. CHO-K1, CHO-DG44, CHO-S, and CHL cells were analyzed in two biological replicates. CHO-K1-IgG, CHL-IgG cells, and Chinese hamster lung tissues were analyzed in three biological replicates.

RNA sequencing (RNA-seq) was conducted using three CHO cell lines: CHO-K1, CHO-DG44, and CHO-S. It was also performed on an IgG1-producing clone derived from CHO-K1 (hereafter referred to as CHO-K1-IgG) and CHL-YN cells (hereafter referred to as CHL cells), along with their IgG1-producing clone, named CHL-IgG.. Cells were harvested during the late logarithmic phase of batch culture, and RNA-seq analysis identified 25,367, 27,664, 26,514, 20,281, 24,048, and 18,372 transcripts from CHO-K1, CHO-DG44, CHO-S, CHO-K1-IgG, CHL, and CHL-IgG cells, respectively, prior to data filtering. Hierarchical clustering and principal component analysis (PCA) were performed based on transcripts per million (TPM) values to evaluate transcriptomic similarity. The TPM dataset was log_2_-transformed with a pseudo-count of 1 added to reduce the influence of genes with high baseline expression on the clustering and PCA. The parental CHL and CHL-IgG cells were most distantly related to the three parental CHO cell lines and CHO-K1-IgG cells (Fig. 1a). CHO-K1 cells exhibited higher similarity to their derivative CHO-K1-IgG than to the other parental CHO lines. These findings suggested that cell lineage (e.g., CHL vs. CHO origin) and clonal relationship (e.g., CHO-K1 and CHO-K1-IgG) exert a greater influence on transcriptomic similarity than whether a cell line is parental or an antibody-producing clone. In PCA, the first principal component (PC1) separated the CHL lineage from the CHO lineage (Fig. 1b). Along PC1 and the second principal component (PC2), CHL cells displayed similar levels of dissimilarity to all three parental CHO cell lines. The differences between the antibody-producing clones and their respective parental cell lines, namely CHL vs. CHL-IgG and CHO-K1 vs. CHO-K1-IgG, were most clearly represented by PC2. The number of differentially expressed genes (DEGs) identified between CHL and each of the CHO parental lines, CHO-K1, CHO-DG44, and CHO-S, were 1,206, 3,311, and 2,861, respectively (Fig. 1c). A total of 3,966 DEGs were found between the antibody-producing clones CHL-IgG and CHO-K1-IgG (Fig. 1d). Overall, 767 DEGs were commonly observed across all three parental cell comparisons and the clone comparison (Fig. 1c,d).

Proteomic analysis was performed using CHL-IgG cells, CHO-K1-IgG cells, and lung tissue derived from Chinese hamster. The CHO-K1-IgG and CHL-IgG cells used for proteomics were collected at the same time as those used for transcriptomics. Using LC–MS/MS, 9,676, 9,633, and 9,479 proteins were identified from CHL-IgG cells, CHO-K1-IgG cells, and lung tissue, respectively, prior to data filtering. To evaluate similarities between cultured cells and lung tissue, hierarchical clustering and PCA were conducted. Protein quantification values were normalized based on total peptide intensity. After filtering using criteria of false discovery rate (FDR) < 0.01 and Mascot score > 40, the values were log_2_-transformed with a pseudo-count of 1 added and used for subsequent analyses. Hierarchical clustering revealed greater differences between lung tissue and cultured cells than between CHO-K1-IgG and CHL-IgG cells (Fig. 1e). PCA showed that PC1 separated lung tissue from the cultured cell lines, while PC2 distinguished between CHO-K1-IgG and CHL-IgG cells (Fig. 1f). Interestingly, lung tissue exhibited comparable levels of similarity to both CHL-IgG cells, which are derived from lung tissue, and CHO-K1-IgG cells, which originate from the same closed colony of Chinese hamsters.

### Enriched biological processes and pathways in transcriptomics of CHL and CHO cells

**Fig. 2:**
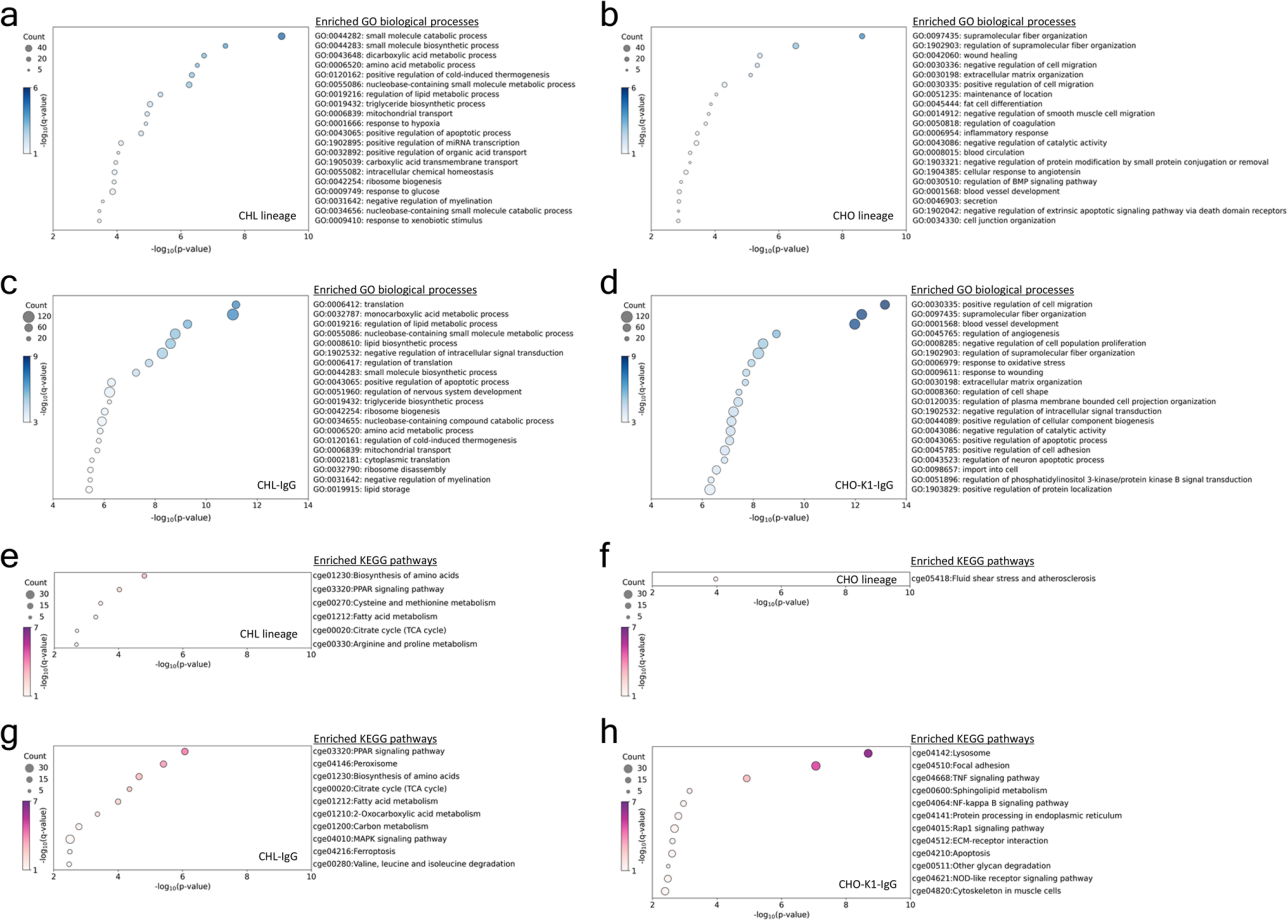
Transcriptomics with GO and KEGG^54,55^ pathway enrichment analyses. Top 20 enriched biological process-related terms in GO enrichment analysis with DEGs upregulated greater than twofold (**a**) in CHL and CHL-IgG cells against parental CHO and CHO-K1-IgG cells, (**b**) in parental CHO and CHO-K1-IgG cells against CHL and CHL-IgG cells, (**c**) in CHL-IgG cells against CHO-K1-IgG cells, and (**d**) in CHO-K1-IgG cells against CHL-IgG cells. Significantly enriched terms in KEGG pathway enrichment analysis with DEGs upregulated greater than twofold (**e**) in CHL and CHL-IgG cells against parental CHO and CHO-K1-IgG cells, (**f**) in parental CHO and CHO-K1-IgG cells against CHL and CHL-IgG cells, (**g**) in CHL-IgG cells against CHO-K1-IgG cells, and (**h**) in CHO-K1-IgG cells against CHL-IgG cells. In the figures, the size of plots, the color of plots, and the x-axis indicate the number of DEGs categorized in each term, FDR transformed using -log_10_, and p-value transformed using -log_10_, respectively. P-values represent the raw statistical significance of enrichment, while q-values indicate the FDR-adjusted significance. Terms with p < 0.01 and q < 0.1 were considered significantly enriched in exploratory analysis. The use of the KEGG database in this study was conducted under authorization granted by Kanehisa Laboratories^54,55^.

From the 767 common DEGs across all pairs, mRNAs showing a fold change in expression greater than 2 for each pair were selected to perform GO enrichment analysis using mRNAs upregulated in the CHL lineage and those upregulated in the CHO lineage. The DEGs used for this analysis are listed in Supplementary Table S1. In the CHL lineage, biological processes related to organic acid metabolism, such as the amino acid metabolic process, dicarboxylic acid metabolic process, and positive regulation of organic acid transport, were upregulated (Fig. 2a). Additionally, metabolic processes associated with nucleobases and lipids were also upregulated. Other important processes related to antibody production, such as those involved in translation and mitochondria, were also upregulated. In the CHO lineage, processes related to maintenance of the cytoskeleton and tissue structure and secretion-related processes were upregulated (Fig. 2b). Among the 3,966 DEGs identified in the comparison between antibody-producing clones, mRNAs showing a fold change greater than 2 were selected to perform GO enrichment analysis with those upregulated in CHL-IgG and CHO-K1-IgG cells. CHL-IgG cells showed upregulation of organic acid, nucleobase, and lipid metabolism (Fig. 2c). Processes involved in the regulation of translation and ribosome assembly were found to be more strongly active. In contrast, in the CHO-K1-IgG cells, processes related to secretion were not among the top 20 enriched terms, unlike in the overall comparison (Fig. 2b,d). Instead, processes related to the cytoskeleton and tissue structure maintenance were more strongly active (Fig. 2d).

Kyoto Encyclopedia Genes and Genome (KEGG)^54,55^ pathway enrichment analysis was performed using the mRNA list previously employed in the GO enrichment analysis. In the comparison across all pairs, the CHL lineage exhibited enrichment in pathways related to amino acid metabolism, such as cysteine and methionine metabolism, as well as arginine and proline metabolism (Fig. 2e). Pathways associated with lipid metabolism, including the PPAR signaling pathway and fatty acid metabolism, were upregulated in alignment with the GO enrichment analysis (Fig. 2a,c,e). Notably, upregulation of the TCA cycle was observed. Conversely, the CHO lineage showed only one pathway that surpassed the q-value threshold, which was related to shear stress (Fig. 2f). In the comparison between antibody-producing clones, the CHL-IgG cells exhibited consistently upregulated pathways related to amino acid biosynthesis, lipid metabolism, and TCA cycle (Fig. 2e,g). In the CHO-K1-IgG cells, protein processing in the endoplasmic reticulum was notably upregulated (Fig. 2h). The lysosome pathway was the most enriched, further supporting that post-translational processes were more active in CHO-K1-IgG cells. Additionally, pathways related to glycosylation, which is crucial for the quality of antibodies as pharmaceuticals, were also upregulated.

### Enriched biological processes and pathways in proteomics of IgG-producing CHL and IgG-producing CHO-K1 cells

**Fig. 3:**
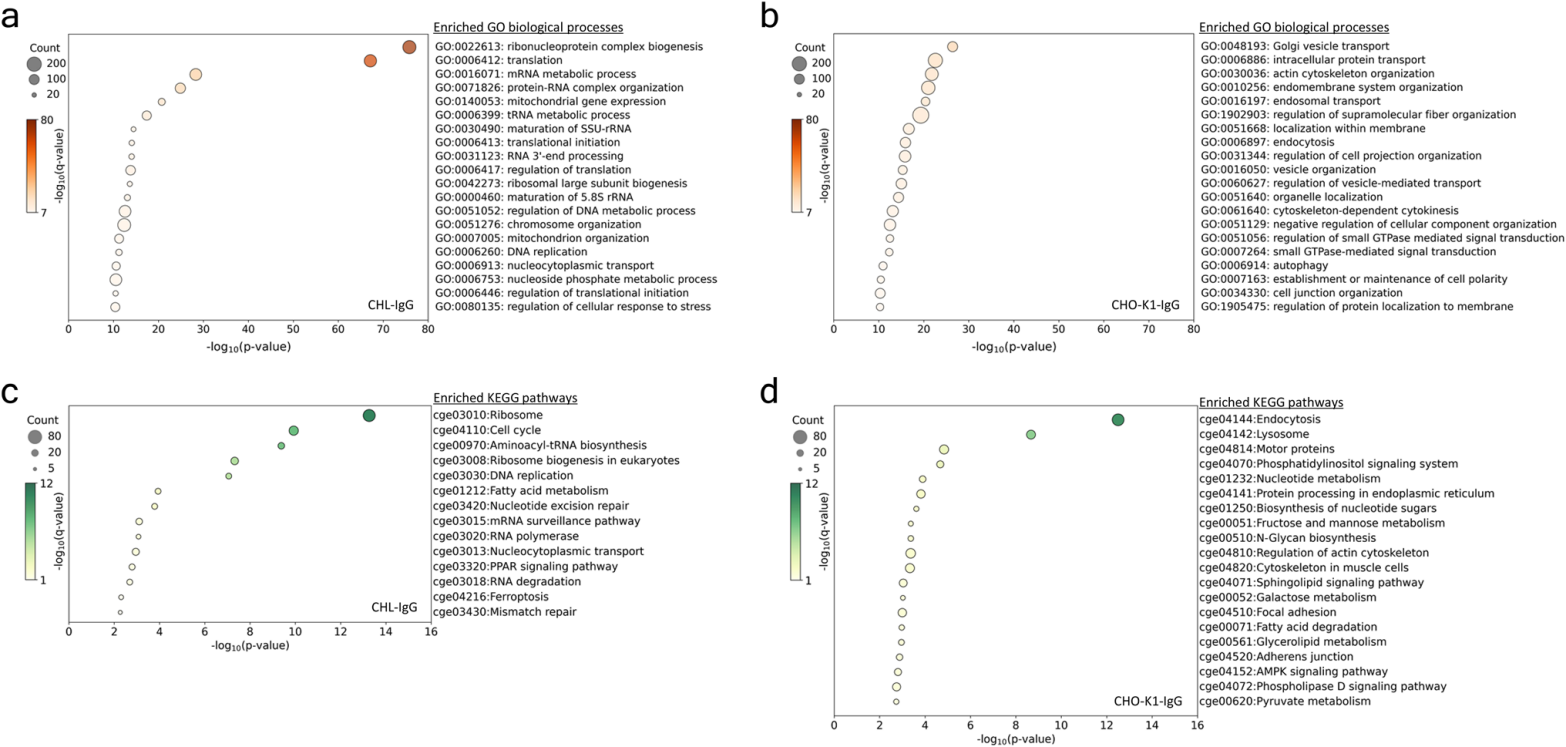
Proteomics with GO and KEGG^54,55^ pathway enrichment analyses. Top 20 enriched biological process-related terms in GO enrichment analysis with DEGs upregulated greater than 1.2 fold (**a**) in CHL-IgG cells against CHO-K1-IgG cells and (**b**) in CHO-K1-IgG cells against CHL-IgG cells. Top 20 enriched pathway terms in KEGG pathway enrichment analysis with DEGs upregulated greater than 1.2 fold (**c**) in CHL-IgG cells against CHO-K1-IgG cells and (**d**) in CHO-K1-IgG cells against CHL-IgG cells. In the figures, the size of plots, the color of plots, and the x-axis indicate the number of DEGs categorized in each term, FDR transformed using -log_10_, and p-value transformed using -log_10_, respectively. P-values represent the raw statistical significance of enrichment, while q-values indicate the FDR-adjusted significance. Terms with p < 0.01 and q < 0.1 were considered significantly enriched in exploratory analysis. The use of the KEGG database in this study was conducted under authorization granted by Kanehisa Laboratories^54,55^.

From the DEGs between CHO-K1-IgG and CHL-IgG cells, proteins with a fold change greater than 1.2 were selected. GO enrichment analysis and KEGG pathway analysis were then performed using the proteins upregulated in CHL-IgG cells and in CHO-K1-IgG cells. Among the top 20 enriched terms in CHL-IgG cells, 13 were related to translation (Fig. 3a). While GO enrichment analysis using transcriptomic data also revealed the enrichment of translation-related processes in CHL-IgG cells, the proteomics showed stronger enrichment in these processes (Fig. 2a,c and 3a). Processes related to mitochondria and nucleobase metabolism were consistently upregulated in CHL-IgG cells, in agreement with the transcriptomics. In contrast to the transcriptomics, proteomics notably revealed the upregulation of processes related to the cell cycle, such as chromosome organization and DNA replication (Fig. 3a). In the CHO-K1-IgG cells, 4 of the top 20 enriched terms were associated with vesicle transport, directly related to antibody secretion (Fig. 3b). Processes related to endocytosis and autophagy were upregulated in CHO-K1-IgG cells, thus, similar to the transcriptomics, post-translational processes were active at the protein level (Fig. 2b,h and 3b).

KEGG pathway enrichment analysis was performed using the same protein list used for the GO enrichment analysis. In CHL-IgG cells, similar to the transcriptomic data, pathways related to fatty acid metabolism and the PPAR signaling pathway were upregulated, demonstrating that lipid metabolism pathways are also highly regulated at the protein level (Fig. 2e,g and 3c). Among the 14 enriched terms, 7 were related to translation, consistent with the GO enrichment analysis, showing that translation-related pathways are strongly active in CHL-IgG cells (Fig. 2a,c and 3a,c). Pathways related to the cell cycle, such as mismatch repair and DNA replication, were represented by 4 of the 14 enriched terms. In CHO-K1-IgG cells, pathways related to endocytosis, lysosome, and protein processing in the ER were upregulated, indicating that post-translational pathways are consistently and highly regulated (Fig. 2h and 3b,d). Among the top 20 enriched terms, 4 were related to lipid metabolism and signaling, including the sphingolipid signaling pathway and glycerolipid metabolism, while 2 terms were associated with nucleobase metabolism (Fig. 3d). These findings revealed that lipid and nucleobase metabolism were not uniformly upregulated in CHL-IgG cells compared with CHO-K1-IgG cells. Additionally, pathways related to glycan metabolism, such as galactose metabolism, were enriched in CHO-K1-IgG cells.

### Lung tissue-derived characteristics in CHL-IgG cells and a closed colony of Chinese hamster-derived characteristics in CHO-K1-IgG cells

**Fig. 4:**
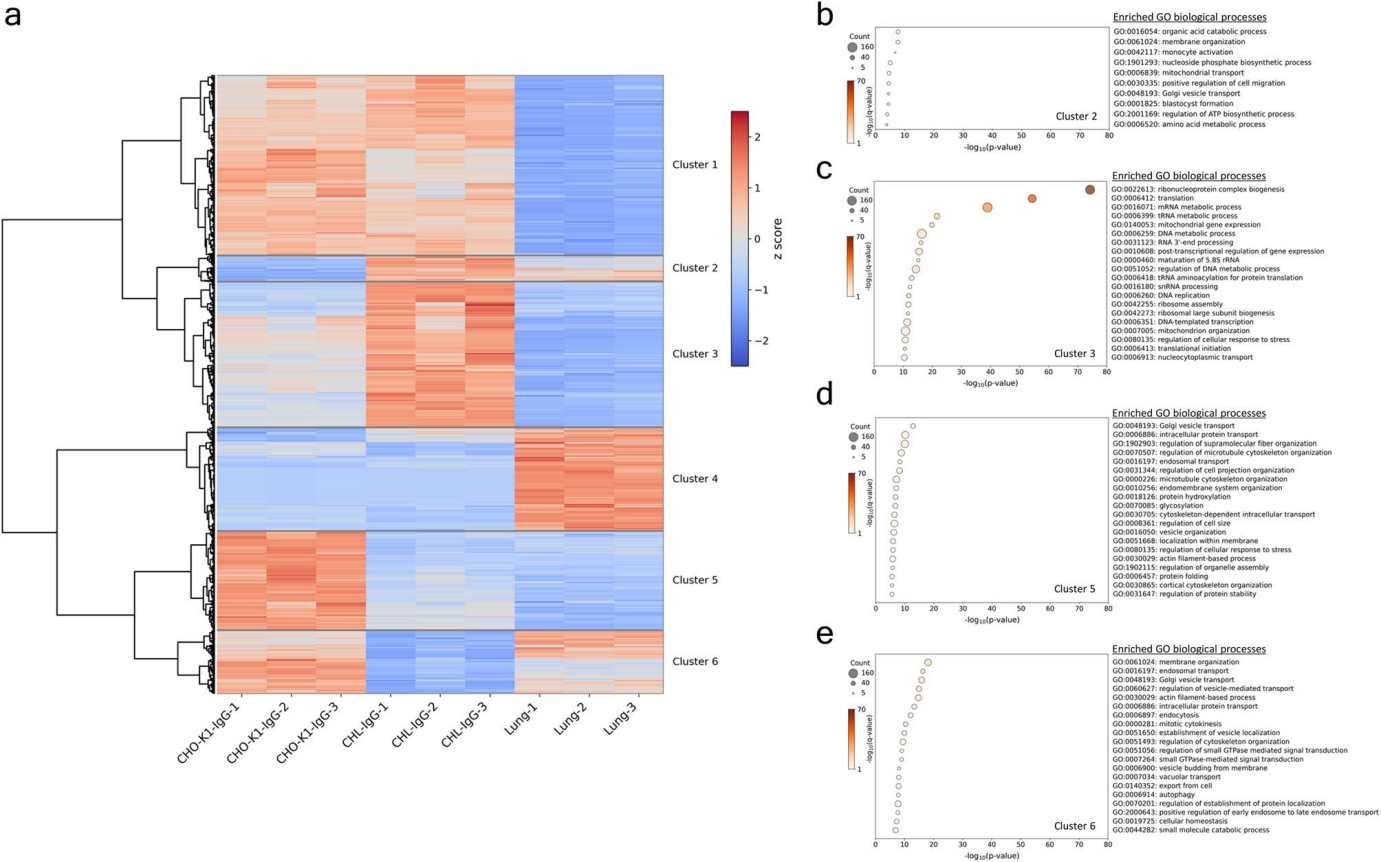
Proteomic commonalities and differences among CHO-K1-IgG, CHL-IgG cells, and lung tissues. (**a**) All DEGs in any pairs among CHO-K1-IgG, CHL-IgG cells, and lung tissues were clustered into six groups by hierarchical clustering with z-score transformed protein expression values. In the heatmap, red indicates upregulation of the genes. (**b**–**e**) Top 20 enriched terms in biological processes with DEGs of Clusters 2, 3, 5, and 6. Clusters 2, 3, 5, and 6 contain (b) DEGs commonly upregulated in CHL-IgG cells and lung tissues, (**c**) DEGs specifically upregulated only in CHL-IgG cells, (**d**) DEGs specifically upregulated only in CHO-K1-IgG cells, and (**e**) DEGs commonly upregulated in CHO-K1-IgG cells and lung tissues, respectively. In the figures, the size of plots, the color of plots, and the x-axis indicate the number of DEGs categorized in each term, FDR transformed using -log_10_, and p-value transformed using -log_10_, respectively. P-values represent the raw statistical significance of enrichment, while q-values indicate the FDR-adjusted significance. Terms with p < 0.01 and q < 0.1 were considered significantly enriched in exploratory analysis.

Quantitative protein values of all DEGs between CHL-IgG cells, CHO-K1-IgG cells, and lung tissue were converted to z-scores, and hierarchical clustering was performed. The clustering resulted in six major clusters, which were classified into three clusters showing high expression exclusively in CHL-IgG cells, CHO-K1-IgG cells, or lung tissue, and three clusters showing high expression in two of the cell types (Fig. 4a). Cluster 2, which was upregulated in CHL-IgG cells and lung tissue, contained fewer proteins than Cluster 6, which was upregulated in both CHO-K1-IgG cells and lung tissue. GO enrichment analysis was performed using the proteins in Clusters 2, 3, 5, and 6 to identify the features of CHL-IgG cells that are derived from lung tissue and unique to CHL-IgG cells, and the features of CHO-K1-IgG cells that are derived from the closed system colony and unique to CHO-K1-IgG cells, respectively. GO enrichment analysis using proteins in Cluster 2 revealed enrichment in organic acid metabolism, including amino acid metabolism, mitochondrial processes involved in ATP biosynthesis regulation, nucleobase metabolism, and vesicle transport (Fig. 4b). In Cluster 3 containing proteins exclusively upregulated in CHL-IgG cells, 14 of the top 20 enriched terms were related to translation, and processes related to mitochondria, nucleobase metabolism, and DNA replication were upregulated (Fig. 4c). Interestingly, translation and cell cycle-related processes were absent from the enriched terms in Cluster 2 (Fig. 4b). This suggests that the translation and cell cycle upregulated in CHL-IgG cells are unique to these cells and not derived from lung tissue. Furthermore, organic acid metabolism, which was characteristic of the CHL lineage in transcriptomics, was upregulated exclusively in Cluster 2, indicating that this feature is likely shared with lung tissue (Fig. 2 and 4b). In Cluster 5 containing proteins exclusively upregulated in CHO-K1-IgG cells, processes related to vesicle transport, endocytosis, protein folding, and glycosylation were enriched (Fig. 4d). Cluster 6 showed enrichment in vesicle transport and endocytosis (Fig. 4e). Pathway enrichment analysis using DEGs at the protein level between CHO-K1-IgG and CHL-IgG cells revealed enrichment in glycosylation and protein processing in the ER in CHO-K1-IgG cells, indicating that these features are unique to CHO-K1-IgG cells. Although not shown in the figures, Cluster 1, which represents processes commonly upregulated in cell lines compared with lung tissue, showed enrichment of processes related to transcription, translation, and the cell cycle, suggesting active cell growth of cell lines. In contrast, Cluster 2, which reflects processes characteristically upregulated only in lung tissue, indicated enrichment for pathways associated with cytoskeletal organization and maintenance of tissue structure, thereby capturing the distinct differences between tissues and cell lines.

### Gene expression in distinctive biological processes and pathways between IgG-producing CHO and IgG-producing CHL cells

**Fig. 5:**
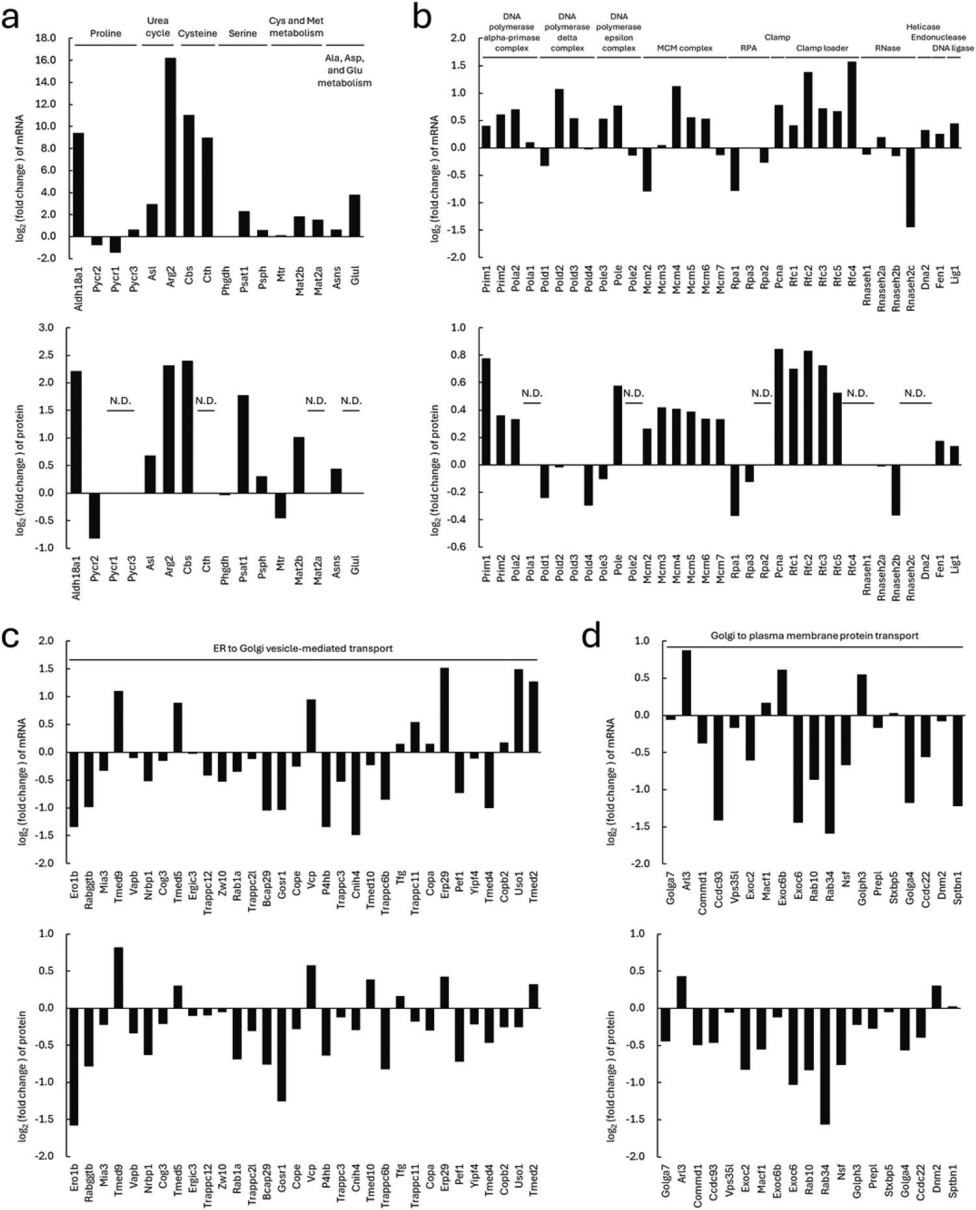
Gene expression in characteristic biological processes and pathways in CHL-IgG and CHO-K1-IgG cells. Log_2_ transformed fold changes between CHL-IgG and CHO-K1-IgG cells of mRNA and protein expression in (**a**) biosynthesis of amino acids, (**b**) DNA replication, (**c**) ER to Golgi vesicle-mediated transport, and (**d**) Golgi to plasma membrane protein transport. Positive values indicate higher expression in CHL-IgG cells, while negative ones indicate higher expression in CHO-K1-IgG cells. N.D. indicates not detected. The protein numbers of N.D. were five in (**a**) and seven in (**b**).

In CHL-IgG cells, pathways related to organic acid metabolism and the cell cycle were upregulated (Fig. 2 and 3). To further investigate these pathways, the expression of genes involved in amino acid biosynthesis and DNA replication was compared between CHL-IgG and CHO-K1-IgG cells. Among the 16 mRNAs involved in amino acid biosynthesis, 14 were highly expressed in CHL-IgG cells, and of the 11 proteins detected, 8 showed higher expression in CHL-IgG cells (Fig. 5a). Notably, enzymes involved in the biosynthesis of cysteine, which has low solubility and stability in chemically defined media for animal cell culture^30,31^, as well as glutamine, such as Cbs, Cth, and glutamine synthetase (Glul), exhibited 2088, 510.3, and 14.15 times higher mRNA expression in CHL-IgG cells than in CHO-K1-IgG cells. In DNA replication, out of 33 genes, 23 were upregulated at the mRNA level, and 18 out of 26 detected proteins were highly expressed in CHL-IgG cells (Fig. 5b). The expression of genes associated with three DNA polymerase complexes, the minichromosome maintenance (MCM) complex (a helicase complex involved in the initiation and progression of DNA replication), and the DNA clamp loader complex (which facilitates the attachment of DNA clamps to template DNA to enable continuous synthesis by DNA polymerase) was particularly high in CHL-IgG cells.

For CHO-K1-IgG cells, which showed significant upregulation of vesicle transport, the expression of genes related to ER to Golgi vesicle-mediated transport and Golgi to plasma membrane protein transport were compared. Of the 32 randomly selected genes involved in ER to Golgi vesicle-mediated transport, 21 were upregulated at both mRNA and protein levels in CHO-K1-IgG cells (Fig. 5c). Among these genes, Ero1b and Gosr1 showing greater than two-fold higher protein expression are involved in disulfide bond formation in the ER and vesicle membrane fusion in the Golgi, respectively. For Golgi to plasma membrane protein transport, of 19 randomly selected genes, 12 showed higher expression at both mRNA and protein levels in CHO-K1-IgG cells (Fig. 5d). Genes upregulated in CHO-K1-IgG cells at the protein level by more than two-fold included Exoc6, a component of the exocyst complex involved in vesicle transport and membrane fusion, and Rab34, a GTPase involved in lysosome positioning and endocytosis.

## Discussion

In this study, the transcriptomic and proteomic profiles of CHL cells, which grow twice as fast as conventional CHO cells, were compared to those of CHO cells and Chinese hamster lung tissue using statistical and bioinformatic analyses. The differences in the overall biological processes and pathways between CHL cells and CHO cells were evaluated, and further investigation identified characteristics of CHL cells derived from lung tissue and those uniquely found in CHL cells.

In hierarchical clustering using the transcriptome, the clusters were divided into two major groups: one for CHL cells and another for CHO cells. PCA similarly separated the two groups based on PC1. Both statistical analyses revealed that CHO-K1-IgG and CHL-IgG cells were most similar to their respective parental cells, CHO-K1 and CHL, indicating that the parental origin of the cell line, rather than the expression of IgG_1_, had a greater impact on the cellular similarity. Hierarchical clustering and PCA using the proteome assessed the similarity between the cell lines and lung tissue. Both statistical methods revealed significant differences between the two cell lines and lung tissue. This was consistent with previous studies using the proteome from various Chinese hamster tissues and two CHO cell lines, which demonstrated a clear separation between cell lines and tissues based on PC1^28^. These findings suggest that the changes occurring during the isolation, adaptation, and modification of cell lines after they are obtained from their tissue source have a greater impact on their similarity than the tissue origin itself.

In transcriptomics, biological processes related to the metabolism of organic acids, nucleobases, and lipids were upregulated in the CHL lineage. Pathway analysis revealed that these processes included cysteine and methionine metabolism, arginine and proline metabolism, and the PPAR signaling pathway. Previous research comparing the metabolomics of CHL and CHO cells during fed-batch culture showed differences in the temporal changes of concentration of metabolites involved in arginine and methionine metabolism, such as putrescine and glutathione, with higher expression of related genes in CHL cells^17^. Putrescine, a polyamine, functions as a growth supplement in CHO-K1 cells^32^, and intracellular glutathione levels have been reported to correlate with antibody productivity in CHO cells^33,34^. Therefore, the upregulation of these amino acid metabolism pathways in CHL cells could offer a potential advantage over CHO cells in biopharmaceutical production. In CHL cells, the TCA cycle, mitochondria, and translation were upregulated. Organic acid metabolism, the TCA cycle, and mitochondria-related processes are all involved in the production of energy essential for cellular activity, while lipid metabolism and translation are associated with biomass production. The active energy and biomass production processes in CHL cells could be necessary for the rapid cell growth. By contrast, lysosome, protein processing in the ER, and glycan metabolism were enriched in the CHO lineage. The upregulation of post-translational processes in CHO cells suggests that these cells possess the ability to efficiently secrete recombinant proteins. Therefore, to expand the potential of CHL cells as biopharmaceutical production hosts, future research should focus on cell engineering to enhance these post-translational processes and the identification of biomarkers for selecting CHL cells with strong post-translational processes. The upregulated shear stress-associated pathway in CHO lineage implies that CHO cells are sensitive to shear stress even in culture with a shake flask compared with CHL lineage, while CHL cells potentially have resistance to shear stress.

In proteomics, translation, cell cycle, and mitochondria-related processes were upregulated in CHL-IgG cells, consistent with the transcriptomics. Especially, the translation-associated processes highly enriched at the protein level suggested that post-transcriptional processes were strongly regulated in CHL-IgG cells. The strength of translation-related processes was characterized in CHL-IgG cells by the upregulation of several processes, including ribosome biogenesis, mRNA processing, and transport of translation-related factors. The gene amplification system in CHO cells increases mRNA expression of recombinant protein genes, which in turn increases translation and secretion. However, these processes are not always linear and can be influenced by the efficiency of translation and post-translational processes^35,36^. CHL cells could have a higher likelihood of avoiding translation bottlenecks in recombinant protein expression compared with CHO cells, which could be advantageous for membrane protein production that does not require secretion. To verify this, it is important to compare the translation efficiency between CHL and CHO cells. Interestingly, processes related to the cell cycle were upregulated in CHL-IgG cells. Among cell cycle-related processes, DNA replication, mismatch repair, and chromosome organization were enriched, suggesting that CHL cells could have stronger functions in genome replication and error correction than CHO cells. This finding is consistent with the fast growth capability of CHL cells. One characteristic of CHO cells is genome instability, which has been reported to lead to inconsistent productivity and quality of recombinant protein^37–39^. Therefore, a deeper understanding of the genome instability in CHL cells could contribute to the development of more stable host cells. In CHO-K1-IgG cells, vesicle transport was particularly enriched. Studies analyzing the secretion process of antibodies in CHO cells have reported that antibodies, once assembled, accumulate in the ER, and thus vesicle transport is one of the bottlenecks in the secretion process^40,41^. Research aimed at improving vesicle transport from the ER to the Golgi has shown that overexpression of lectin proteins such as ERGIC-53 and the GTPase Sar1a involved in COPII vesicle formation enhanced antibody productivity in CHO cells^42,43^. Therefore, routinely achieving antibody yields exceeding 10 g L^−1^ and specific productivity greater than 20 pg cell^−1^ day^−1^ could be attributed to the strong vesicle transport and other secretion processes in CHO cells. In the pathways of CHO-K1-IgG cells, protein processing in the ER and glycan metabolism, directly involved in antibody production, were upregulated. In protein processing in the ER, overexpression of PDI and a signal receptor protein Srp14 has been shown to improve folding and assembly efficiency, and enhance antibody productivity^44,45^. Additionally, the galactosylation of *N*-glycosylation on antibodies improves the complement-dependent cytotoxicity activity of therapeutic antibodies. Therefore, the upregulated protein processing in the ER and glycan metabolism in CHO cells underscore why CHO cells are the major host for therapeutic antibody production.

To identify the features of CHL-IgG cells that are derived from lung tissue and those unique to CHL cells, as well as to distinguish the features of CHO-K1-IgG cells that are derived from the same closed colony and those unique to CHO cells, the proteomes of CHL-IgG, CHO-K1-IgG cells, and lung tissue were separated into six clusters by hierarchical clustering. Biological processes related to energy production were specifically upregulated in Cluster 2, while biological processes related to translation and the cell cycle were upregulated only in Cluster 3. Thus, some of the activated energy production processes in CHL-IgG cells could be derived from lung tissue. Previous studies analyzing the proteomic profiles of two CHO cell lines and several Chinese hamster tissues showed that translation-related processes were enriched in the heart, while cell cycle-related processes were enriched in CHO-S cells^28^. To further investigate whether the upregulated translation and cell cycle-related processes in CHL-IgG cells are unique, a comparison with CHO-S cells or heart tissue would be valuable. In the comparison of Clusters 5 and 6, it was found that the upregulated protein folding and glycosylation processes in CHO-K1-IgG cells are likely unique to these cells. Previous studies reported that vesicle transport processes are enriched in the ovaries and lung tissue^28^, suggesting that the upregulated vesicle transport in CHO-K1-IgG cells could be partially derived from the ovaries. Cluster 2 showed upregulated Golgi vesicle transport; thus, the strong vesicle transport derived from lung tissue may be somewhat maintained in CHL-IgG cells.

The expression levels of genes involved in amino acid biosynthesis and DNA replication as well as genes related to ER to Golgi vesicle-mediated transport and Golgi to plasma membrane protein transport were compared at both mRNA and protein levels. Cysteine is a precursor of the antioxidant glutathione, and cysteine deficiency can lead to oxidative stress^46^. However, cysteine forms dimers due to its low stability and solubility in neutral pH, leading to precipitation in culture medium. To overcome this, attempts have been made to overexpress enzymes involved in cysteine biosynthesis in CHO cells, enabling them to synthesize cysteine^30^. CHO cells engineered to acquire cysteine biosynthetic capacity exhibited comparable growth to conventional fed-batch cultures even under cysteine-depleted fed-batch conditions, and demonstrated enhanced tolerance to oxidative stress, thereby indicating the potential to improve antibody production^30^. Among these, the mRNA expression of Cbs and Cth was higher in CHL-IgG cells than in CHO-K1-IgG cells, potentially reducing the requirement for cysteine in the culture medium. Therefore, it is important to investigate whether CHL cells possess sufficient endogenous capacity to biosynthesize cysteine. The mRNA expression of Glul was high in CHL-IgG cells. Glutamine is sensitive to temperature and unstable in medium, and thus it is generally added just before use. The high expression of Glul in CHL-IgG cells suggests their potential to be cultured in media without glutamine, simplifying cell culture operations. Genes related to DNA replication were generally highly expressed in CHL-IgG cells. For cells to grow rapidly, the genome must be replicated quickly and accurately. The high expression of genes involved in mismatch repair, nucleotide excision repair, and DNA replication could support the high growth capacity of CHL cells. The expression of genes related to ER to Golgi vesicle-mediated transport and Golgi to plasma membrane protein transport was generally higher in CHO-K1-IgG cells than in CHL-IgG cells. Numerous studies have identified various bottlenecks in the pathway from the ER to the Golgi and made improvements in this regard^40,42–45^. Therefore, rather than focusing on single processes through cell engineering to enhance secretion, an approach that enhances the entire secretory pathway could be more effective. Cell engineering strategies that reprogram the secretion process, such as overexpressing Blimp1, involved in differentiation into secretory plasma cells, and Xbp1, which activates chaperones, may be particularly effective in CHL cells^47^. Downregulation of processes such as vesicle transport leads to inefficiencies in the secretory process, whereas their upregulation has been reported to partially reduce cell growth rate^43,47^. Therefore, upregulation of vesicle transport in CHL cells may potentially impose a limitation on high cell growth capacity.

Here, omics profiling of CHL cells, CHO cells, and Chinese hamster lung tissue revealed that processes related to energy and biomass production, as well as the cell cycle, are active in CHL cells, while post-translational processes are active in CHO cells. Furthermore, characteristics of these cell lines are partly derived from the respective tissues. CHL cells are believed to have advantages as biopharmaceutical production hosts, particularly in translation, cell cycle processes, and metabolic pathways. The most distinctive feature of CHL cells, namely their approximately two-fold faster growth rate compared with conventional CHO cells, holds the potential to reduce by half the time typically required—around one year each—for both the screening of high-producing clones and the scale-up of cell cultures. A deeper understanding of their unique features could shorten the research and development timelines for biopharmaceutical production, as well as expand options for selecting cell lines tailored to specific process objectives. Moreover, elucidating the mechanisms underlying the high growth capacity of CHL cells could lead to improvements in the growth abilities of traditional CHO cells.

## Methods

### Cell culture

CHO-K1, CHO-K1-IgG cells derived from IgG_1_-producing CHO-K1 cells, and CHL and CHL-IgG cells derived from IgG_1_-producing CHL cells were grown in EX-CELL CD CHO Fusion supplemented with 6 mM L-glutamine (FUJIFILM Wako Pure Chemical Corporation, Osaka, Japan). CHO-K1-IgG, CHL, and CHL-IgG cells were previously described as CHO-HcD6^48^, CHL-YN^16^, and CHL-YN-IgG1 B-08 cell clone^17^, respectively. CHO-S and CHO-DG44 cells were grown in FreeStyle CHO Expression Medium (Thermo Fisher Scientific, Waltham, MA, USA) supplemented with 6 mM L-glutamine　and in EX-CELL 302 Serum-Free Medium for CHO Cells (Sigma Aldrich, St. Louis, MO, USA) supplemented with 6 mM L-glutamine. Batch cultures were performed in 125 mL Erlenmeyer shake flasks (Corning, Corning, NY, USA) at 37 °C, 5% CO_2_, 80% humidity, and 90 rpm with an orbital diameter of 50 mm for CHO-K1, CHO-S, and CHO-DG44 cells, and 140 rpm with an orbital diameter of 25 mm for CHO-K1-IgG, CHL, and CHL cells on a Climo-Shaker ISF1-XC (Kühner, Birsfelden, Switzerland). Viable cell concentration and cell viability were measured using a Vi-CELL Cell Viability Analyzer (Beckman Coulter, Brea, CA, USA). Cell samples containing 3 × 10^5^ to 5 × 10^5^ cells for RNA-seq and proteomics were collected late in the exponential growth phase, which was 48 h after culture initiation for CHO-S, 54 h for CHL and CHL-IgG cells, 72 h for CHO-K1 and CHO-DG44 cells, and 90 h for CHO-K1-IgG cells, when the viability was higher than 90%. The culture supernatant was removed by centrifugation, and the cell pellets were stored at − 80 °C until use.

### Tissue harvesting

Lung tissue was harvested from 7-week-old female Chinese hamster derived from the same closed colony as the original Chinese hamster that was the source of the currently most utilized CHO cell lines^28,49^. In accordance with prior proteomic analyses of Chinese hamster-derived tissues and cell lines, lung tissue was harvested at the same postnatal age as reported in these studies^28,29^. The approximate body weight of the hamster used for this study was 26.8 g. This value was calculated from the wet weight of the harvested lung, 182 mg, based on the reported relationship between the body weight and the wet weight of lung of mice^50^. The approximate body weight of the hamster was estimated based on mouse data, and interspecies differences between mouse and Chinese hamster should be recognized as a limitation. All of the animal experiments were conducted in accordance with all guidelines and regulations of Johns Hopkins University Animal Care and Use Committee (Approved Protocol HA21E192). All experimental protocols were approved by Johns Hopkins University Animal Care and Use Committee. This study was reported in accordance with the ARRIVE guidelines (https://arriveguidelines.org). Euthanasia was performed using CO_2_ gas and cervical dislocation. Immediately after harvesting, the lung tissue was washed three times with ice-cold PBS, divided into small pieces, frozen with dry ice, and stored at − 80 °C until use.

### RNA-seq

mRNA was extracted from each cell line using RNeasy Mini Kit (Qiagen, Germantown, MD, USA). Two biological replicates were used for parental cells, including CHO-S, CHO-DG44, CHO-K1, and CHL cells. Three biological replicates were used for IgG-producers, including CHO-K1-IgG and CHL-IgG cells. The quality of the total RNA was checked using Qsep 100 DNA Fragment Analyzer (BiOptic, San Francisco, CA, USA) for CHO-S, CHO-DG44, and CHO-K1 cells, Agilent 2100 Bioanalyzer Instrument (Agilent Technologies, Santa Clara, CA, USA) for CHL cells, and Agilent 4200 TapeStation System (Agilent Technologies) for CHO-K1-IgG and CHL-IgG cells. The RNA-seq library was prepared with KAPA Stranded mRNA Seq Kit (KAPA Biosystems, Wilmington, MA, USA) for sequencing with 76 bp pair-end reads on NextSeq 500 (Illumina, San Diego, CA, USA) for CHO-S, CHO-DG44, CHO-K1, and CHL cells. For CHO-K1-IgG and CHL-IgG cells, the RNA-seq library was prepared with TruSeq Stranded mRNA LT Sample Prep Kit (Illumina) for sequencing with 101 bp pair-end reads on NovaSeq X (Illumina).

### RNA-seq data processing

Raw RNA-seq data were trimmed to remove adapter sequences to filter low-quality sequences by Fastp v0.22.0^51^. The quality of the trimmed sequences was checked by FastQC v0.12.1. The trimmed sequences were mapped on the reference Chinese hamster transcriptome database, GCF_003668045.3_CriGri-PICRH-1.0_rna.fna.gz, by Salmon v1.10.0^52^ to obtain a TPM dataset. Differentially expressed genes (DEGs) between CHO-S and CHL cells, CHO-DG44 and CHL cells, CHO-K1 and CHL cells, and CHO-K1-IgG and CHL-IgG were extracted from the TPM dataset with R studio-driven TCC-GUI^53^. Parameters used in TCC-GUI computation were 10 as the threshold for filtering low-count genes, DESeq2 for normalization and DEG identification methods, and FDR < 0.05.

### Protein preparation for LC–MS/MS

Biological triplicate lung tissues frozen in liquid nitrogen were ground with a motor and pestle until they became powder. The powdered tissues were lysed using RIPA buffer (Thermo Fisher Scientific) supplemented with 1 mM phenylmethylsulfonyl fluoride (PMSF). Biological triplicate cell line samples of CHO-K1-IgG and CHL-IgG cells were lysed with RIPA buffer (Thermo Fisher Scientific) supplemented with 1 mM PMSF. The lysates of lung tissues and cell lines were sonicated on ice until the solution became transparent, followed by the removal of cell debris by centrifugation at 10,000 rpm and 4°C. The concentration of the extracted protein was measured by BCA assay. Then, 50 μg of each extracted protein was dried and reconstituted in 50 μL of 50 mM triethylammonium bicarbonate (TEAB). Next, 12 μL of 15 mg mL^−1^ dithiothreitol (DTT) in 100 mM TEAB was added and incubated for 1 h at 58 °C to reduce the protein. A total of 1.2 μL of 36 mg mL^−1^ Iodoacetamide in 100 mM TEAB was added and incubated for 30 min in the dark to alkylate the protein. Protein purification was performed using SP3 beads (PreOmics, Planegg, Germany) in line with the manufacturer’s instructions, followed by proteolysis on the beads with 100 μL of 27 μg mL^−1^ trypsin (Thermo Fisher Scientific) in 100 mM TEAB at 37 °C with shaking at 1000 rpm overnight. The digested peptides were tandem mass tag (TMT)-labeled on the beads with TMT10plex™ Isobaric Label Reagents and Kits (Thermo Fisher Scientific) in 42 μL of acetonitrile, in accordance with the manufacturer’s instructions. The TMT-labeled peptides were combined, cleaned up on Pierce Det removal columns (Thermo Fisher Scientific), and fractionated by basic reverse-phase liquid chromatography (RPLC) into 24 fractions for LC–MS/MS.

### LC–MS/MS

Each of the 24 fractions was reconstructed in 50 µL of 2% acetonitrile/0.1% formic acid. Then, 3 μL of each fraction was analyzed on a nano-LC-Orbitrap-Lumos (Thermo Fisher Scientific) interfaced with a NEO nano-LC (Thermo Fisher Scientific). The peptides were separated by RPLC on a 75 µm × 150 mm ProntoSIL-120–5-C18 H column 3 µm, 120 Å (BISCHOFF, Turvey, UK), for elution by linearly increasing the ratio of solvent B (0.1% formic acid/90% acetonitrile) to solvent A (0.1% formic acid/2% acetonitrile) over 90 min. Eluted peptides were sprayed into an Orbitrap Fusion Lumos mass spectrometer (New Objective, Littleton, MA, USA) through a 1 µm emitter tip at a spray voltage of 1.9 kV. Full MS survey scans were acquired on an Orbitrap within 400−1500 m/z using a Data-Dependent Top 15 method with dynamic exclusion of 45 s. Precursor ions were individually isolated with 0.7 Da and fragmented (MS/MS) using an HCD activation collision energy of 38. Precursor and fragment ions were analyzed at resolutions of 120,000 and 50,000, respectively.

### Proteomic data processing

Peptide sequences were identified from isotope-resolved mass of MS and MS/MS spectra using Proteome Discoverer 3.2.5 (Thermo Fisher Scientific) and Mascot 2.8.2 (Matrix Sciences, Chicago, IL, USA) based on the *Cricetulus griseus* protein database. Carbamidomethyl on cysteine, TMT6pl on the N-terminus, and TMT6pl on lysine were set as fixed modifications, while oxidation on methionine, and deamidation on asparagine and glutamine were set as variable modifications. Mass tolerance on precursor and product ions was 5 and 10 ppm, respectively. The quantity of identified proteins in each sample was normalized by total peptide amount. Proteins showing low-quality annotation in the NCBI *Cricetulus griseus* database were omitted. Proteins with FDR < 0.01, Mascot Score > 40, and detected in all samples were used as reliable proteins for analysis. DEGs between CHL-IgG and CHO-IgG-K1 cells, CHL-IgG cells and lung tissues, and CHO-K1-IgG and lung tissues were determined at a threshold of p < 0.05 by one-way analysis of variance (ANOVA).

### Transcriptomics

Non-coding mRNA and mRNA showing low-quality annotation in the NCBI *Cricetulus griseus* database were omitted from the DEGs to obtain genes of interest for GO enrichment analysis, KEGG^54,55^ pathway enrichment analysis, and quantitative gene expression comparison. For GO enrichment analysis and KEGG pathway enrichment analysis, the DEGs with more than twofold change in expression were obtained from the common DEGs identified across all of the pairs, including CHO-S and CHL cells, CHO-DG44 and CHL cells, CHO-K1 and CHL cells, and CHO-K1-IgG and CHL-IgG cells. The DEGs between CHO-K1-IgG and CHL-IgG cells with more than twofold changes in expression were separately analyzed as well. mRNA accession numbers were mapped to gene symbols through the NCBI *Cricetulus griseus* database.

GO enrichment analysis was performed for biological processes using Metascape v3.5 (https://metascape.org/gp/index.html)^56^. The conditions used in Metascape were as follows: GO Biological Processes as the ontology source and all genes in the *Mus musculus* genome as the enrichment background. The threshold for collection and grouping was set as p < 0.01 in the cumulative hypergeometric distribution, a minimum count of 3, and an enrichment factor higher than 1.5. Significantly enriched terms were exploratorily determined at q < 0.1 by Benjamini–Hochberg (BH)-corrected FDR^57^.

KEGG pathway enrichment analysis was performed using Database for Annotation, Visualization, and Integrated Discovery (DAVID) v2023q4 (http://www.david.niaid.nih.gov)^58^. The conditions used in DAVID through KEGG pathway enrichment analysis were KEGG_PATHWAY as the annotation category and all genes in the *Cricetulus griseus* genome as the enrichment background. The threshold for collection and grouping was set as p < 0.01 determined with EASE Score^59^, which was a modified Fisher’s exact p-value, and a minimum count of 2. Significantly enriched terms were determined at q < 0.1 by BH-corrected FDR. The formal permission to use the KEGG pathway database in this study was kindly provided by the Kanehisa Laboratories.

### Proteomics

Proteins showing low-quality annotation in the NCBI *Cricetulus griseus* database were omitted from the DEGs to obtain genes of interest for GO enrichment analysis, KEGG pathway enrichment analysis, and quantitative gene expression comparison. When a twofold change was applied as the filtering threshold, as in the transcriptomic analysis, the number of proteins obtained was more than 60% lower than that analyzed in the transcriptomics. To avoid underestimating proteomic data, a 1.2-fold change threshold—corresponding to a comparable level of protein filtering—was employed, and the DEGs exceeding this threshold between CHO-K1-IgG and CHL-IgG cells were used for GO enrichment analysis and KEGG pathway enrichment analysis. Additionally, the quantitative values of all of the DEGs between CHL-IgG and CHO-IgG-K1 cells, CHL-IgG cells and lung tissues, and CHO-K1-IgG and lung tissues were transformed into z-scores to perform hierarchical clustering by Ward’s method^60^. GO enrichment analysis was performed using four of the six distinct clusters. Two clusters exhibited high protein expression exclusively in either CHL-IgG or CHO-K1-IgG cells, while the other two clusters showed high protein expression commonly in CHL-IgG cells and lung tissues or CHO-K1-IgG cells and lung tissues. All of the conditions and parameters used in GO enrichment analysis and KEGG pathway enrichment analysis were the same as those used in the transcriptomics.

### mRNA and protein expression comparison

TPM data and quantitative values of proteins were used for mRNA and protein expression comparisons. For mRNA and proteins containing several variants in their sequence, the most abundant variants for each mRNA and protein were used for the comparison. Gene lists in particular pathways and biological processes were downloaded from the *Cricetulus griseus* database in KEGG (https://www.genome.jp/kegg/pathway.html) and *Mus musculus* database in AmiGO (http://www.geneontology.org)^61^, respectively. Gene lists for pathways of biosynthesis of amino acids and DNA replication were from cge01230 and cge03030, and gene lists for biological processes of ER to Golgi vesicle-mediated transport and Golgi to plasma membrane protein transport were from GO:0,006,888 and GO:0,043,001.

### Statistics and computation

Hierarchical clustering using Ward’s method and PCA with TPM data and quantitative values of proteins were performed in Python v3.12. To reduce the influence of differences of baseline expression levels among genes on the analysis results, the expression values were scaled by applying a log_2_ transformation with the addition of 1. The scaled values were subsequently used for hierarchical clustering and PCA.

## Supplementary Information

Supplementary Information.

## Data Availability

The RNA-seq datasets generated and analyzed during the current study are available in the DDBJ Sequence Read Archive repository with identifier　PRJDB35724 (https://ddbj.nig.ac.jp/search/entry/bioproject/PRJDB35724). The proteome datasets generated and analyzed during the current study are available in the ProteomeXchange Consortium via the PRIDE partner repository with identifier PXD066507 (https://www.ebi.ac.uk/pride/archive?keyword=PXD066507%20).
